# Interactions of the Immune System with Human Kidney Organoids

**DOI:** 10.3389/ti.2024.12468

**Published:** 2024-04-18

**Authors:** Anusha S. Shankar, Hector Tejeda-Mora, Zhaoyu Du, Quincy Nlandu, Virginia Palomares-Cabeza, Thierry P. P. van den Bosch, Sander S. Korevaar, Fabiany Da Costa Gonçalves, Eric M. J. Bindels, R. Kramann, Marlies E. J. Reinders, Marian C. Clahsen-van Groningen, Ewout J. Hoorn, Joost Gribnau, Carla C. Baan, Martin J. Hoogduijn

**Affiliations:** ^1^ Department of Internal Medicine, Erasmus MC, Erasmus MC Transplant Institute, University Medical Center, Rotterdam, Netherlands; ^2^ Department of Oral and Maxillofacial Surgery, Erasmus MC, University Medical Center, Rotterdam, Netherlands; ^3^ Department of Pathology, Erasmus MC, University Medical Center, Rotterdam, Netherlands; ^4^ Department of Hematology, Erasmus MC, University Medical Center, Rotterdam, Netherlands; ^5^ Institute of Experimental Medicine and Systems Biology, Medical Faculty, RWTH Aachen University, Aachen, Germany; ^6^ Division of Nephrology and Clinical Immunology, Medical Faculty, RWTH Aachen University, Aachen, Germany; ^7^ Department of Internal Medicine, Erasmus MC, University Medical Center, Rotterdam, Netherlands; ^8^ Department of Developmental Biology, Erasmus MC, Oncode Institute, University Medical Center, Rotterdam, Netherlands

**Keywords:** immunity, kidney, organoids, implantation, stem cells

## Abstract

Kidney organoids are an innovative tool in transplantation research. The aim of the present study was to investigate whether kidney organoids are susceptible for allo-immune attack and whether they can be used as a model to study allo-immunity in kidney transplantation. Human induced pluripotent stem cell-derived kidney organoids were co-cultured with human peripheral blood mononuclear cells (PBMC), which resulted in invasion of allogeneic T-cells around nephron structures and macrophages in the stromal cell compartment of the organoids. This process was associated with the induction of fibrosis. Subcutaneous implantation of kidney organoids in immune-deficient mice followed by adoptive transfer of human PBMC led to the invasion of diverse T-cell subsets. Single cell transcriptomic analysis revealed that stromal cells in the organoids upregulated expression of immune response genes upon immune cell invasion. Moreover, immune regulatory PD-L1 protein was elevated in epithelial cells while genes related to nephron differentiation and function were downregulated. This study characterized the interaction between immune cells and kidney organoids, which will advance the use of kidney organoids for transplantation research.

## Graphical Abstract

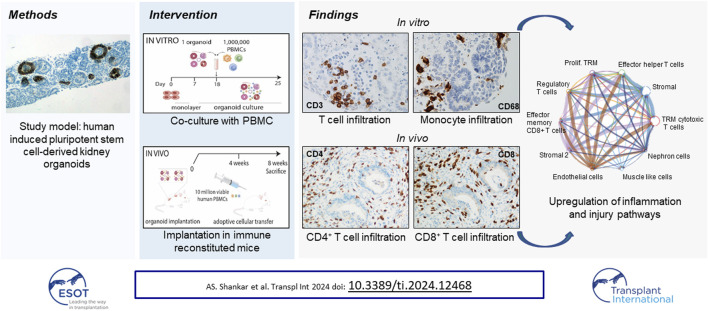

## Introduction

Induced pluripotent stem cell (iPSC)-derived kidney organoids have an ever increasing impact on kidney research through their ability to model kidney disease, their use as a drug screening tool, and their potential application in regenerative medicine [1–4]. They may also be a tool in transplantation research. Kidney organoids represent an early stage of nephrogenesis [5, 6], but nevertheless contain the major kidney structures. Implantation of kidney organoids enhances maturation and function [7–9]. Nephrons within implanted kidney organoids have the potential to function as active filtration units due to the presence of a size-selective glomerular barrier and the active uptake of low molecular weight compounds by the tubular cells [4].

So far, kidney organoid implantations have been performed in immune deficient animal models to avoid immune responses. However, understanding the interaction between immune cells and kidney organoids is important for modelling kidney disease, which frequently has an immunological component, and for transplantation research. Furthermore, the interaction of the immune system with kidney organoids becomes relevant when kidney organoids become tools for kidney regeneration. Ideally, in a clinical setting autologous kidney organoids are used to ensure acceptance by the adaptive immune system. It is however complex and costly to generate patient-specific kidney organoids on demand due to the time-consuming and labor-intensive nature of the procedure and the use of off-the-shelf allogeneic organoids would largely simplify applications with kidney organoids.

Implantation of other PSC-derived cell types such as neurons, cardiomyocytes and pancreatic cells has been shown to evoke immune responses [10–12]. It is unknown whether iPSC-derived kidney organoids behave in a similar manner, or whether they would accommodate the immune system. In addition to adaptive immune responses, cultured cells and organoids may be targets for innate immune responses, which are triggered by culture-induced changes in cellular gene expression profiles.

We investigated the interactions between human immune cells and human kidney organoids in an *in vitro* co-culture system and a subcutaneous implantation mouse model.

## Materials and Methods

### iPSC Culture

Four human iPSC lines were generated from 3 anonymous donors (2 female and 2 clones from a male donor). The age of the donors were 6 and 42 years, and one was unknown. The iPSC lines, derived from healthy primary skin fibroblasts, were reprogrammed by a single, multicistronic lentiviral vector encoding POU5F1, SOX2, KLF4, and C-MYC [13], as described earlier [14]. Written informed consent was obtained from the donors in accordance with the Medical Ethics Committee of the Erasmus University Medical Center (MEC-2017-248) [15]. The studies with the iPSC lines were performed in accordance with the ethical standards as laid down in the 1964 Declaration of Helsinki and its later amendments. Cells were plated on Geltrex (Gibco, Waltham, United States) and cultured in Essential 8 medium (Thermo Fisher Scientific, Bleiswijk, Netherlands).

### Kidney Organoid Differentiation

As adapted from Takasato et al. [16] and our earlier publication [14], iPSC were plated at a density of 20,000–25,000 cells/cm^2^ and cultured in STEMdiff APEL2 medium (APEL2) (STEMCELL Technologies, Vancouver, Canada) supplemented with 8 µM CHIR99021 (R&D Systems, Minneapolis, United States), 5% Protein Free Hybridoma Medium II and 1% Antibiotic-Antimycotic (both Thermo Fisher Scientific) for 3-4 days. Hereafter, 200 ng/mL recombinant human FGF9 (R&D Systems) and 1 μg/mL heparin (Sigma Aldrich, Zwijndrecht, Netherlands) were added until day 7. 500,000 cells were transferred as a pellet onto a 0.4 μm pore polyester transwell membrane (Corning, New York, United States) at an air–liquid interphase and treated for 1 h with 5 μM CHIR99021. Organoids were stimulated with 200 ng/mL recombinant human FGF9 and 1 μg/mL heparin for 5 days, followed by 13 days culture without growth factors. Medium was refreshed every other day.

### PBMC Isolation

Human peripheral blood mononuclear cells (PBMC) were obtained from 2 untyped buffy coats and from 2 blood samples of untyped healthy controls by density gradient centrifugation using Ficoll-Paque (GE Healthcare, New Jersey, United States) and frozen at −80°C. This study was performed according to the tenets of the Declaration of Helsinki and approved by the Medical Ethical Committee of the Erasmus MC (METC-2018-1623).

### Immune Cell-Organoid Co-culture

Two days after removal of growth factors, kidney organoids were transferred to round-bottom polypropylene tubes (Thermo Fisher Scientific). Human PBMC were thawed and added at 1 × 10^6^ cells/organoid in 1 mL APEL medium. Half of the medium was refreshed every other day until day 25. Organoids infiltrated with PBMC were gently washed to remove loosely attached PBMC. Organoids were snap-frozen for gene expression analysis or fixed in 4% paraformaldehyde for 30 min.

### Animal Experiments

Kidney organoids were subcutaneously implanted into 4 dorsal pockets (2 per pocket) in 8–12 weeks old BALB/c IL2Ry^−/−^RAG2^−/−^ immune deficient female mice from own breeding (BALB/cAnNCr originating from Charles River Laboratories, Wilmington, MA, USA). Organoids were implanted with 50 µL Geltrex, as described earlier [14]. One month after organoid implantation, 1 × 10^7^ PBMC were administered via intraperitoneal injection. One month after PBMC administration, mice were sacrificed and blood, spleens and organoids harvested. Spleens and organoids were snap-frozen at −80°C for gene expression analysis, fixed in 4% paraformaldehyde for immunohistochemistry or dissociated into single cells for flow cytometric analysis and/or single cell RNA sequencing. Kidney organoids were minced, digested for 30 min with Collagenase A (3 mg/mL) (Roche) and 10 mM DNase (Sigma Aldrich) and passed through a 70 μm cell strainer (Sigma Aldrich). Heparinized whole blood and cell suspensions of spleens and organoids were stained with anti-mouse CD45-FITC, anti-human CD45-PerCP, CD3-Amcyan, CD4-PE, CD8-APC, CD25-PE-Cy7, CD69-BV421, CD103-PE-Cy7 (All BD Biosciences, Massachusetts, United States) and LIVE/DEAD™ Fixable Red Dead Cell Stain Kit (Thermo Fisher Scientific) for 30 min at 4°C and measured on a FACSCanto II flow cytometer. The experiments were approved by the Dutch Animal Ethical Committee and the local Erasmus University Medical Center Animal Ethical Committee (license number AVD101002016635).

### Real-Time qPCR

mRNA from iPSC and organoids was extracted using the High Pure RNA Isolation Kit (Roche Life Sciences, Indianapolis, United States). cDNA was produced from 500 ng mRNA with random primers (Promega Benelux B.V.). Gene expression was quantified using human-specific TaqMan Gene Expression Assays-on demand (Applied Biosystems) and normalized to GAPDH or CD45.

### (Immuno) Histochemistry

Four-micron sections of FFPE organoids were stained with hematoxylin and eosin (H&E) or Periodic acid-Schiff (PAS). Immunohistochemistry was performed with an automated, validated, and accredited staining system (Ventana Benchmark ULTRA, Ventana Medical Systems, Tucsen, AZ, United States) using ultraview or optiview universal DAB detection Kit. Following deparaffinization and heat-induced antigen retrieval, slides were incubated with the antibody of interest (Supplementary Table S1), followed by hematoxylin II counter stain for 12 min and a blue coloring reagent for 8 min according to the manufacturer’s instructions. For PAS staining, slides were heated to 75°C and diastase added for 12 min, followed by Schiff reagents (Ventana) for 20 min. Finally a counter stain with hematoxylin was performed.

### Quantification of Immunohistochemical Staining

Quantification of immune cell infiltration in kidney organoids was performed by digital evaluation of sections. Stained slides were scanned with the NanoZoomer 2.0-HT and stained areas for different immune cell markers were quantified with QuPath 0.4.1 software. The total area of each organoid was determined and an automatic threshold was generated to determine the positive area in µm^2^. Statistical analysis was performed using two-tailed unpaired Kruskal-Wallis test with Dunn’s multi-comparison test using GraphPad Prism version 8.0.0 (GraphPad Software, La Jolla, CA).

### Multiplex Immunofluorescent Staining

Co-staining of CD45 and Ki67 was performed by automated multiplex IF using the Ventana Benchmark Discovery (Ventana). Following deparaffinization and heat-induced antigen retrieval with CC1 (#950-500, Ventana) for 32 min, slides were incubated with Ki67 for 32 min at 37°C followed by detection with Red610 (#760-245, Ventana). Antigen denature was performed using CC2 (#950-123, Ventana) for 8 min at 100°C. Secondly, CD45 was incubated for 32 min at 37°C followed by detection with FAM (#760-243, Ventana). Slides were washed in PBS and covered with DAPI in vectashield.

### Single Cell RNA Sequencing

Three control organoids (from 2 iPSC lines) and 4 organoids exposed to PBMC (2 iPSC lines) were harvested 1 month after PBMC administration. Organoids were digested by TrypLE Select and stored on ice until sequencing. Libraries were prepared using a Chromium Single Cell Library Kit V3 (10x Genomics, Pleasanton, CA). Next-generation sequencing (28-8-0-91 cycles) was performed on an Illumina NovaSeq 6000 platform (Illumina, San Diego, CA). The raw data was processed into FASTQ files. Raw sequences were inspected for their quality using FastQC (version 0.11.5, Babraham Bioinformatics, Cambridge UK). Reads containing sequence information were mapped to the GRCh38 human genome and mm10 mouse genome (barnyard) reference. Generation of BAM files and filtered gene-barcode matrices was accomplished by Cell Ranger Software (version 6.0 10X Genomics, Pleasanton, CA). The datasets have been deposited to the Sequence Read Archive (SRA) data repository under accession number GSE211803.

### Single Cell RNA Sequencing Analysis

Analysis was performed with Seurat (v4.0.5) [17]. Initial pre-processing removed cells with fewer than 200 distinct transcripts and genes that were expressed in less than three cells and mitochondrial and ribosomal genes were filtered out. Data was normalized for sequencing depth by dividing by the total number of UMIs in every cell and then transformed to a log scale for each cell using the NormalizeData function. Normalized data was then scaled to have the same mean across cells and standard deviation of 1 using the ScaleData function. Principle component analysis was performed using the 10,000 most variable genes identified; principle components were found significant when they reached at least 2x standard deviation. Cells were clustered using the shared-nearest-neighbor modularity optimization-based clustering algorithm with a resolution of 0.5. Uniform-manifold-approximation-projection (UMAP) was used for the 2-dimensional representation of the data. Cells that co-expressed human and mouse genes were filtered out using single cell doublet scoring [18].

### Cluster Identification

Lists of differentially expressed genes between clusters were generated using the Seurat: FindAllMarkers function with a log-fold change above 0.25. Clusters containing both human and mouse genes were discarded for further analysis. A total of 48,235 cells and 68,383 genes (human and mouse) was used for cluster identification. Annotation of clusters was performed by analyses of the expression of representative genes [9, 14].

Representative genes used for identification of non-immune cell clusters are shown in Supplementary Figure S1 (complete list shown in Supplementary Table S1). For the three control samples 348 nephron cells were identified in total, and for the four organoids with immune cells 736 nephron cells were found.

For human immune cells, gender-related genes were driving cell sub-clustering. Therefore samples were integrated using anchor genes using canonical correlation analysis. The machine learning tool CellTypist [19] was used for immune cell subset classification. Visualizations using the DotPlot function graphically represent per cluster percentage expression by dot diameter and average nonzero expression in log2 scale by dot color; data was not shown if the percentage of expression was lower than 0.01.

### Pathway Analysis

Differential pathway analysis was performed for control organoids and organoids with immune cells using GO Biological Processes 2021 and differences between groups were identified using a Wilcoxon rank sum test with a *p*-value cut off of 0.05 for differentially expressed genes. Analysis of cell-cell communication networks was performed using Hill-Langmuir equations implemented in CellChat [20]. The method for calculating the average gene expression per cell cluster was trimean. The threshold of calculated *p*-values was 0.05. All other parameters were kept as default.

### Ligand-Receptor Analysis

Intercellular communication inference was performed with CellChat based on the validated ligand-receptor database [20]. All human cell types identified in the dataset were used for the analysis; mouse cells were excluded. Briefly, from the significantly different expressed genes, ligand-receptor pairs were identified across all cell types and their probability/strength was determined using the trimean method.

### Statistical Analysis

The Mann-Whitney test and the Kruskal-Wallis test with the Dunn post-hoc test were used for comparisons between 2 or more than 2 groups, respectively. Statistical significance was considered for *p* values less than 0.05 (*), 0.01 (**) and 0.001 (***). Data analysis was performed using GraphPad Prism version 8.0.0 (GraphPad Software, La Jolla, CA).

## Results

### Immune Cells Infiltrate Kidney Organoids *in vitro*

**FIGURE 1:**
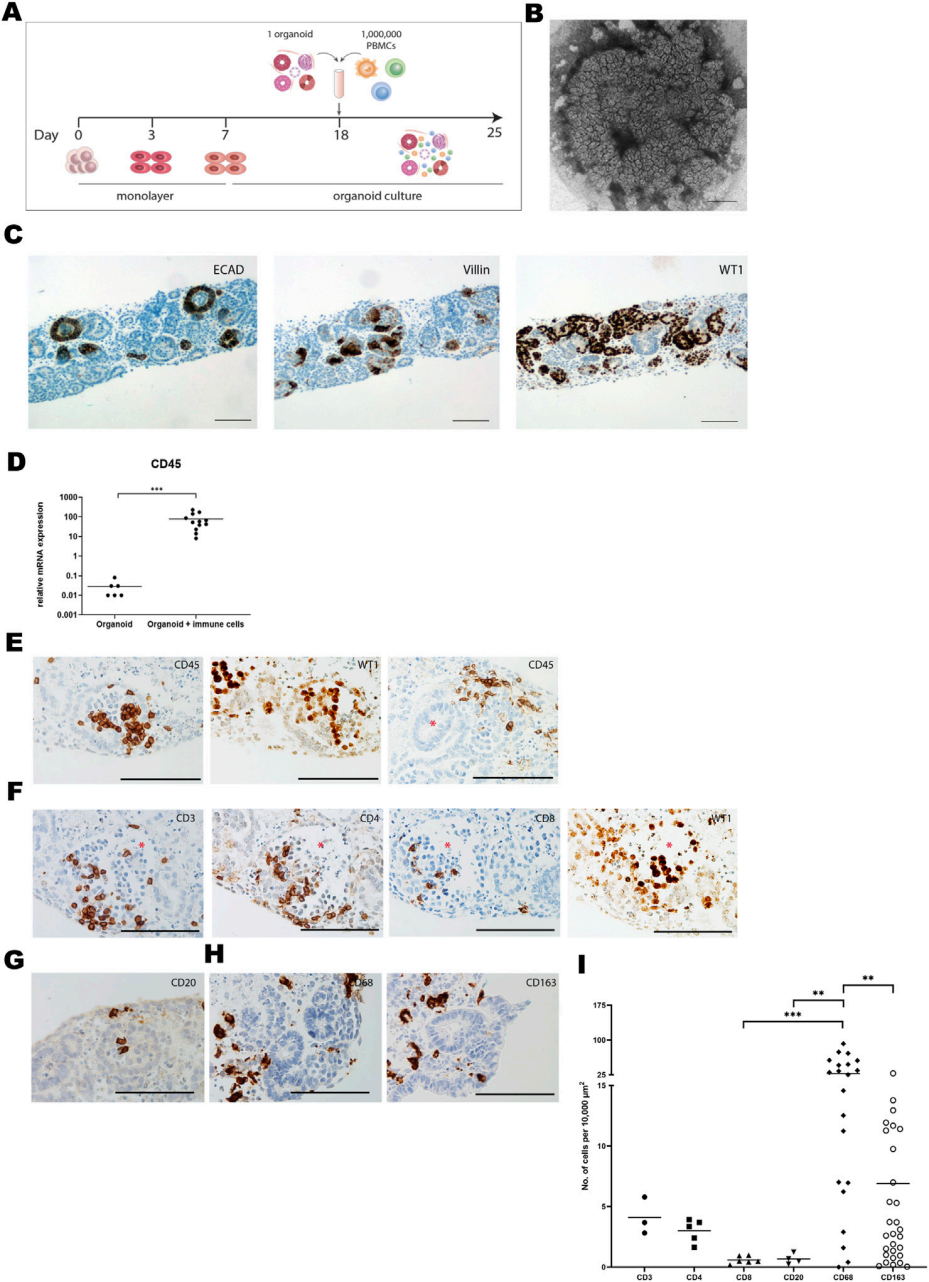
Immune cells infiltrate kidney organoids *in vitro*. **(A)** Schematic drawing of the organoid-PBMC co-culture. **(B)** Brightfield image of an organoid (cultured without immune cells) harvested at day 25 of differentiation. Scale bar indicates 1 mm. **(C)** Expression of the distal tubular marker ECAD, the proximal tubular marker Villin and the glomerular marker WT1 in kidney organoid sections at day 25 of differentiation (without immune cells). Scale bars represent 100 µm. **(D)** mRNA expression of CD45 in organoid-PBMC co-culture compared to only organoid. Data is expressed as ratio with GAPDH x 1000 with SE (n = 12). **(E)** Immunohistochemistry for CD45 depicting infiltration of CD45^+^ leukocytes in glomerular (left) and stromal structures (right). Asterisk marks a tubular structure. Scale bar represents 100 µm. **(F)** Immunohistochemistry for CD3, CD4 and CD8 in consecutive slides showing the distribution of CD4^+^ and CD8^+^ T-cells (asterisk marks a glomerular structure). Scale bar represents 100 µm. **(G)** Immunohistochemistry for CD20, revealing a rare presence of B-cells. Scale bar represents 100 µm. **(H)** Immunohistochemistry for CD68 showing presence of macrophages in the interstitial space. CD163 staining shows the presence of regulatory macrophages. Scale bar represents 100 µm. **(I)** Comparison of the number of infiltrated cells for various immune cell subsets through quantification of immunohistochemical staining by image analysis.

A kidney organoid-PBMC co-culture system was established by culturing kidney organoids in submerged conditions together with allogeneic PBMC for 7 days (Figure 1A). In the absence of PBMC, kidney organoids displayed structural organization at day 25 of culture (Figure 1B) and expressed ECAD, Villin-1, and WT1, indicative of distal tubular, proximal tubular and glomerular structures, respectively (Figure 1C), as demonstrated before [14]. Upon co-culture with PBMC, leukocytes infiltrated into the organoids, evidenced by a 1000-fold mRNA increase of the common leukocyte marker CD45 (Figure 1D). CD45^+^ leukocytes mainly infiltrated WT1^+^ glomerular structures and the stromal compartment without invading tubular structures (Figure 1E). We next examined the subset composition of infiltrated leukocytes and their localization. CD3^+^ T-cells were found within the glomeruli, and CD4^+^ T-cells were more abundant than CD8^+^ T-cells (Figure 1F). CD20^+^ B-cells were scarce (Figure 1G). CD68^+^ macrophages were present in clusters in the interstitial space of the organoids, and we found a subset of CD163^+^ regulatory macrophages (Figure 1H). CD68^+^ macrophages were the most abundant immune cell subset in the organoids, and approximately 25% expressed the regulatory marker CD163 (Figure 1I). CD3^+^ T-cells were 7 times less abundant than CD68^+^ macrophages, and the majority was CD4^+^.

### Immune Cells Infiltrated in *in vitro* Organoids Show an Activated Status

**FIGURE 2:**
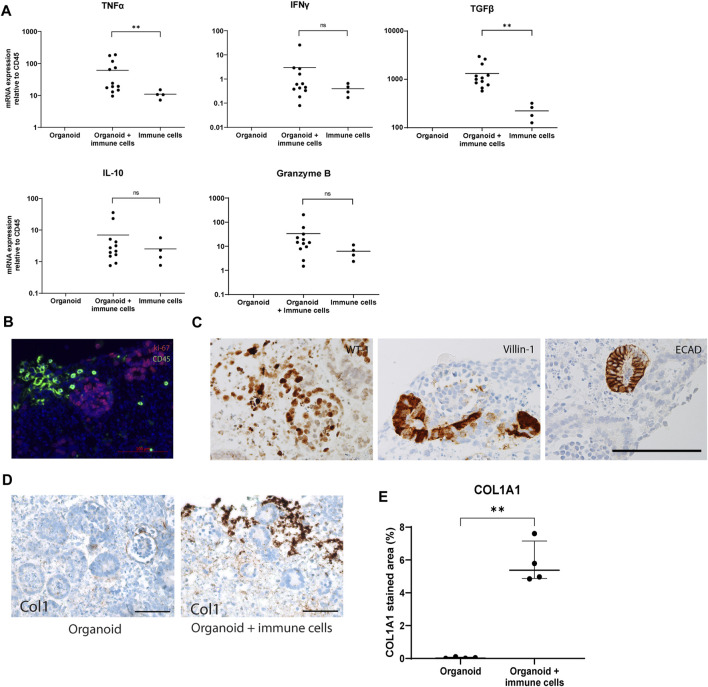
Weak activation status of immune cells infiltrated in *in vitro* organoids. **(A)** Gene expression analysis of inflammatory cytokines. Data is expressed as ratio with GAPDH x 1000 with SE (*n* = 12). **(B)** Double immunofluorescence staining for CD45 (green) and Ki-67 (red) showing absence of proliferating leukocytes. Scale bar represents 100 µm. **(C)** Immunohistochemistry for differentiation markers WT1, Villin-1 and ECAD showing maintenance of differentiation in the organoids exposed to immune cells. Scale bar represents 100 µm. **(D)** Immunohistochemistry for collagen type 1 in organoids cultured with and without immune cells. Scale bar represents 100 µm. **(E)** Collagen 1 type 1 (Col1A1) quantification in immunohistochemical staining in organoids cultured with and without immune cells.

Next, the activation status of infiltrating immune cells was studied in the co-culture system. Gene expression analysis showed a 6-fold increase in mRNA of TNFα and TGF-β in organoids with infiltrated immune cells compared to PBMC cultured without organoids, while IFNγ, IL-10 and Granzyme B did not show any significant differences in their expression levels (Figure 2A). Organoids cultured without immune cells showed no expression of the measured cytokines, suggesting they were immune cell-derived, or produced by the organoids in response to their interaction with immune cells. A double staining for CD45 and Ki67 demonstrated that the infiltrated immune cells were not proliferating (Figure 2B), and kidney organoids maintained their expression of differentiation markers WT-1, Villin-1, and ECAD in the presence of infiltrated immune cells (Figure 2C). Collagen type 1 expression was robustly upregulated in the outer edges of organoids cultured with immune cells (Figures 2D, E). These results indicate that immune cells invade kidney organoids *in vitro*, do not induce major inflammatory responses, but induce fibrotic processes within the 7-day time frame of the experiments.

### Immune Cells Infiltrate Vascularized Kidney Organoids

**FIGURE 3:**
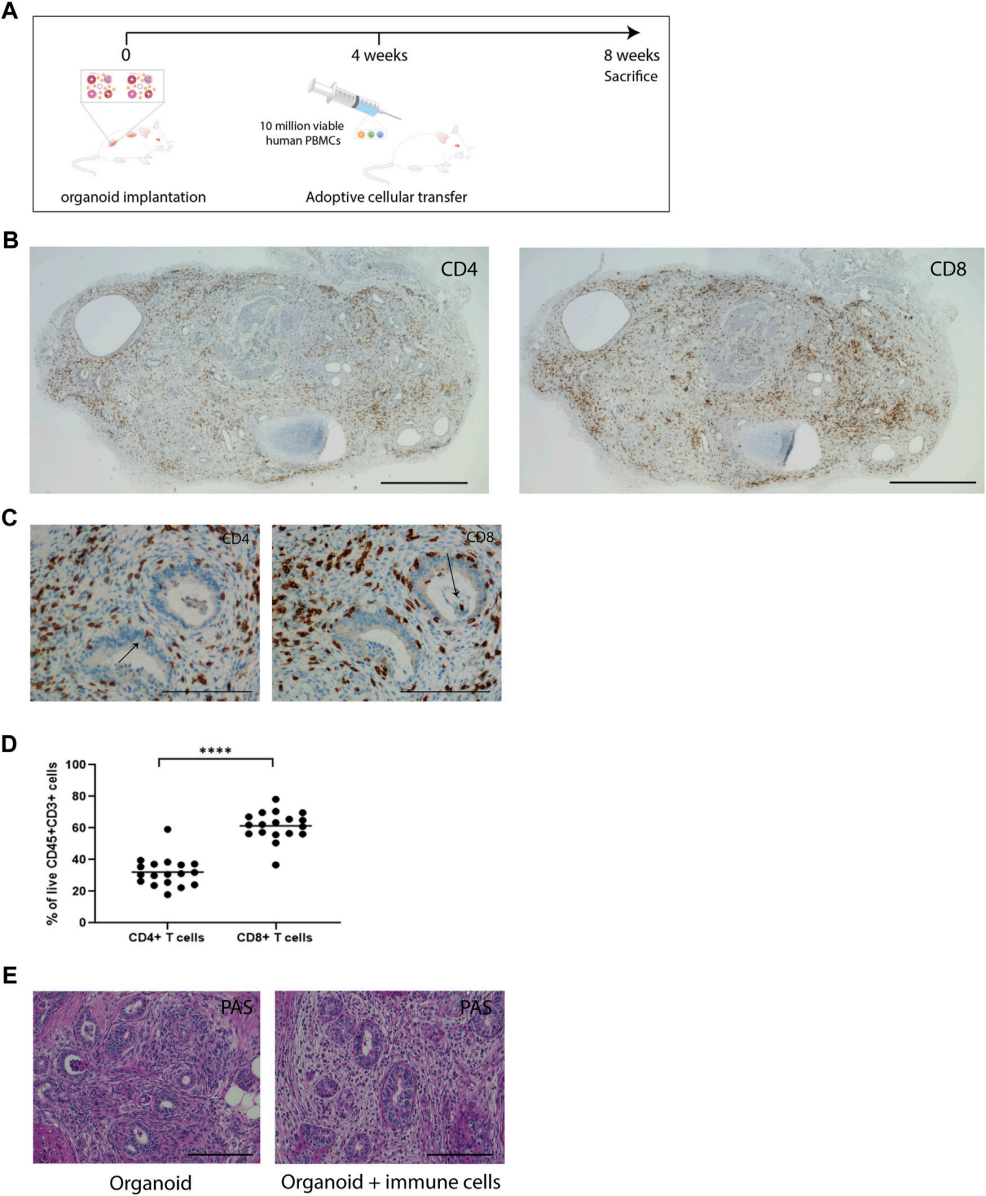
Immune cells infiltrate vascularized implanted kidney organoids. **(A)** Timeline of *in vivo* organoid implantation model and adoptive transfer of PBMC. **(B)** Immunohistochemical staining of CD4 and CD8 in immune cell-infiltrated whole organoids. Scale bar represents 2 mm. **(C)** Immunohistochemistry for CD4 and CD8 in consecutive slides in implanted organoids, showing infiltration of immune cells in all compartments of the organoids. Arrows indicate CD4 and CD8 staining in nephron structures. Scale bars represent 100 µm. **(D)** Proportion of CD4^+^ and CD8^+^ T-cells in organoids determined by flow cytometry (*n* = 18 organoids from 11 mice in 4 individual experiments). **(E)** Representative Periodic acid-Schiff (PAS) staining of implanted organoids retrieved from animals without immune cell administration (organoid) and from animals that received immune cells (organoid + immune cells). Scale bars represent 100 µm.

As the *in vitro* co-culture system does not allow for long-term survival of PBMC and, due to the submerged culture conditions limits oxygen supply to the inner areas of the kidney organoids leading to the development of a necrotic core, we studied long-term interactions between immune cells and kidney organoids in an immune deficient IL2Ry^−/−^RAG2^−/−^ mouse model. Subcutaneously implanted kidney organoids engraft and vascularize successfully in this model [14]. Vascularized kidney organoids differ from their *in vitro* counterparts as a model for alloreactivity chiefly in the route of infiltration of immune cells, which enter from the vascular network in vascularized organoids and from the exterior in *in vitro* organoids. In addition, implanted organoids are 8 weeks older than the *in vitro* organoids at the moment of analysis. During this period, the number of stromal cells in the interstitial space expands and thus the cellular composition of the organoids changes. We administered human PBMC 4 weeks after organoid implantation and analyzed organoids, spleens and blood 4 weeks later (Figure 3A).

#### Effects on Blood and Splenic Phenotype

Spleen weights increased 2- and 6-fold in mice that received PBMC and organoids or PBMC only, respectively, compared to controls (Supplementary Figure S2A). This difference may be explained by the attraction of immune cells toward the organoids, leading to a lower engraftment of immune cells in the spleen. Flow cytometric analysis showed that administration of PBMC with or without organoid implantation resulted in a similar engraftment of human CD45^+^ cells in blood (16% and 15%) and spleens (60% and 55%) (Supplementary Figures S2B, S3). The engrafted immune cell subsets comprised only of CD4^+^ and CD8^+^ T-cells (Supplementary Figure S4), in accordance with previous publications [21]. CD8^+^ T-cells were more abundant than CD4^+^ T-cells in blood and spleen (Supplementary Figure S2C). Interestingly, the presence of organoids had an impact on the CD4^+^/CD8^+^ ratio and increased the frequency of CD4^+^ T cells and decreased the CD8^+^ T cell frequency.

#### Effects on Kidney Organoids

In the organoids, on average 70% of infiltrated CD45^+^ leukocytes were of human origin (Supplementary Figures S2D, S5). Immunohistochemical analysis of implanted organoids demonstrated robust infiltration of CD4^+^ and CD8^+^ cells (Figure 3B). The implanted organoids exhibited a pattern of tubulitis through the presence of CD4^+^ and CD8^+^ T-cells in the basolateral aspect of the tubular epithelial cells (Figure 3C). T-cells also accumulated in the interstitial space between the nephron structures. Flow cytometric analysis of dissociated organoids demonstrated that CD8^+^ cells were more abundant than CD4^+^ cells (61% ± 2.1% vs. 32% ± 2.1%) (Figure 3D), which mimicked the CD4/CD8 ratio in blood and spleen. PAS staining of implanted organoids demonstrated fibrotic tissue in the interstitium (Figure 3E).

### Composition and Activation Status of Infiltrated T-cells

**FIGURE 4:**
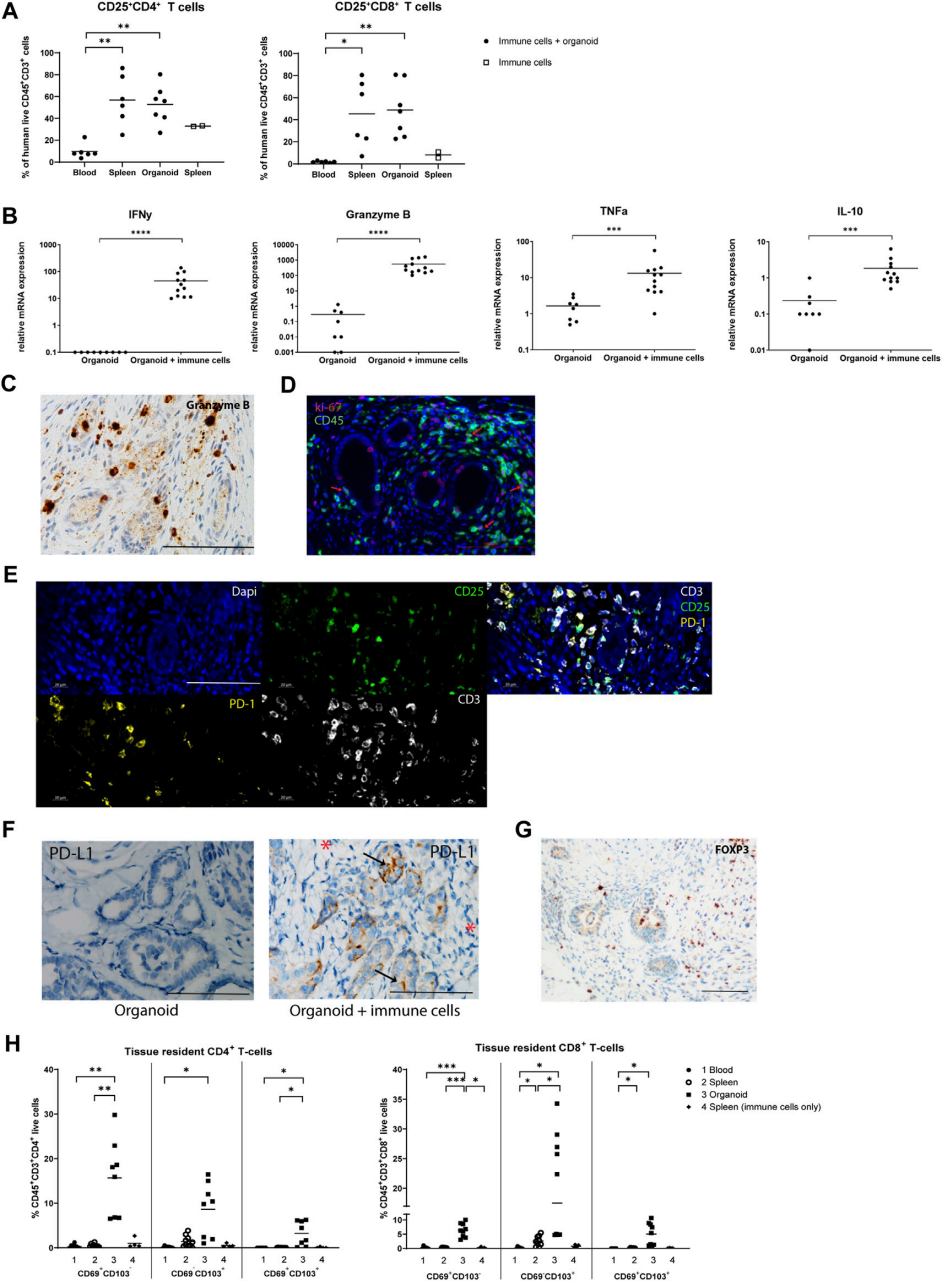
Composition and activation status of infiltrated T-cells in implanted organoids. **(A)** Proportion of activated CD25^+^CD4^+^ T-cells and CD25^+^CD8^+^ T-cells in blood, spleen, and organoids of animals that received immune cells determined by flow cytometry (*n* = 6 or 7 mice from 2 individual experiments). The proportion of activated CD25^+^CD4^+^ T-cells and CD25^+^CD8^+^ T-cells in spleens of animals that received immune cells only (no organoid) is shown as control (*n* = 2 from 2 individual experiments). **(B)** mRNA gene expression analysis of inflammatory cytokines, indicating the initiation of an immune response in organoids with immune cells. Data is expressed as ratio with GAPDH x 1000 with SE (*n* = 12). **(C)** Immunohistochemistry for Granzyme B^+^ cells, revealing Granzyme B^+^ cells surrounding tubular structures and in the interstitial space. Scale bar represents 100 µm. **(D)** Double immunofluorescence staining for CD45 (green) and Ki-67 (red) showing presence of proliferating leukocytes (red arrows). Scale bar represents 100 µm. **(E)** Triple immunofluorescence staining for CD3 (white), CD25 (green) and PD-1 (yellow) showing that many of the infiltrated CD3^+^ T-cells display CD25 expression and many show co-staining with PD-1. Panels from left to right, top to bottom: nuclear Dapi staining; CD25 staining; CD3^−^CD25-PD-1 triple staining; PD-1 staining; CD3 staining. Scale bar represents 100 µm. **(F)** Immunohistochemistry for PD-L1 in immune cell-infiltrated organoids (left panel) showing expression in epithelial cells (arrows) but not in the stromal areas (asterisks). Organoids without immune cells show no PD-L1 expression (right panel). Scale bar represents 100 µm. **(G)** Immunohistochemistry for FOXP3 demonstrates the presence of T-cells with a regulatory phenotype. Scale bar represents 100 µm. **(H)** Proportion of tissue resident CD4^+^ T-cells and CD8^+^ T-cells in the blood, spleen, and organoids of animals that received immune cells determined by flow cytometry (*n* = 8 mice from 2 individual experiments). The proportion of tissue resident CD4^+^ T-cells and CD8^+^ T-cells in spleens of animals that received immune cells only (no organoid) is shown as control (*n* = 4 mice from 2 individual experiments).

We next examined the activation status of infiltrated T-cells by measurement of the expression of the activation marker CD25. The frequency of CD25^+^CD4^+^ and CD25^+^CD8^+^ T-cells was increased approximately 5- and 20-fold in spleens and organoids compared to blood, indicating that infiltrated T-cells have an elevated activation status, or, alternatively, have a regulatory function (Figure 4A). In contrast to *in vitro* organoids, implanted organoids infiltrated by immune cells exhibited elevated mRNA expression of pro-inflammatory IFNy, Granzyme B and TNFα, and anti-inflammatory IL-10, pointing to ongoing immune processes (Figure 4B). Granzyme B^+^ cells were observed surrounding tubular structures and in the interstitial space (Figure 4C). CD45 and Ki67 double immunofluorescence demonstrated the presence of proliferating leukocytes, albeit at a low frequency (Figure 4D). Triple immunofluorescence for CD3, CD25, and PD-1 showed that several of the infiltrated CD3^+^ T-cells stained positive for CD25, suggesting either an activated state or a regulatory function. Many of the infiltrated CD3^+^ T-cells that expressed programmed death protein 1 (PD-1), associated with immune-regulation and immune tolerance (Figure 4E). Interestingly, the ligand for PD-1 (PD-L1) was induced in epithelial cells of kidney organoids upon infiltration with immune cells, while stromal cells were negative (Figure 4F). In addition, a number of cells expressed FOXP3 protein (Figure 4G), which suggests that in addition to immune activating processes, immune regulatory activities are ongoing in the organoids.

### Tissue Resident T-cells in Kidney Organoids

T-cells that reside non-transiently in tissues adapt a tissue-resident phenotype, characterized by CD69 and/or CD103 expression. These non-migratory cells have been shown to play a role in the defense against pathogens, but also to act as mediators of allograft rejection [22]. We found that CD4^+^ and CD8^+^ tissue-resident subsets CD69^+^CD103^−^, CD69^−^CD103^+^ and CD69^+^CD103^+^ were increased in organoids compared to spleens and blood, indicating that the organoids stimulate infiltrated T-cells to adopt a tissue-resident phenotype (Figure 4H; Supplementary Figure S6).

### Single Cell Transcriptomic Analysis of Kidney Organoids and Infiltrated Immune Cells

**FIGURE 5:**
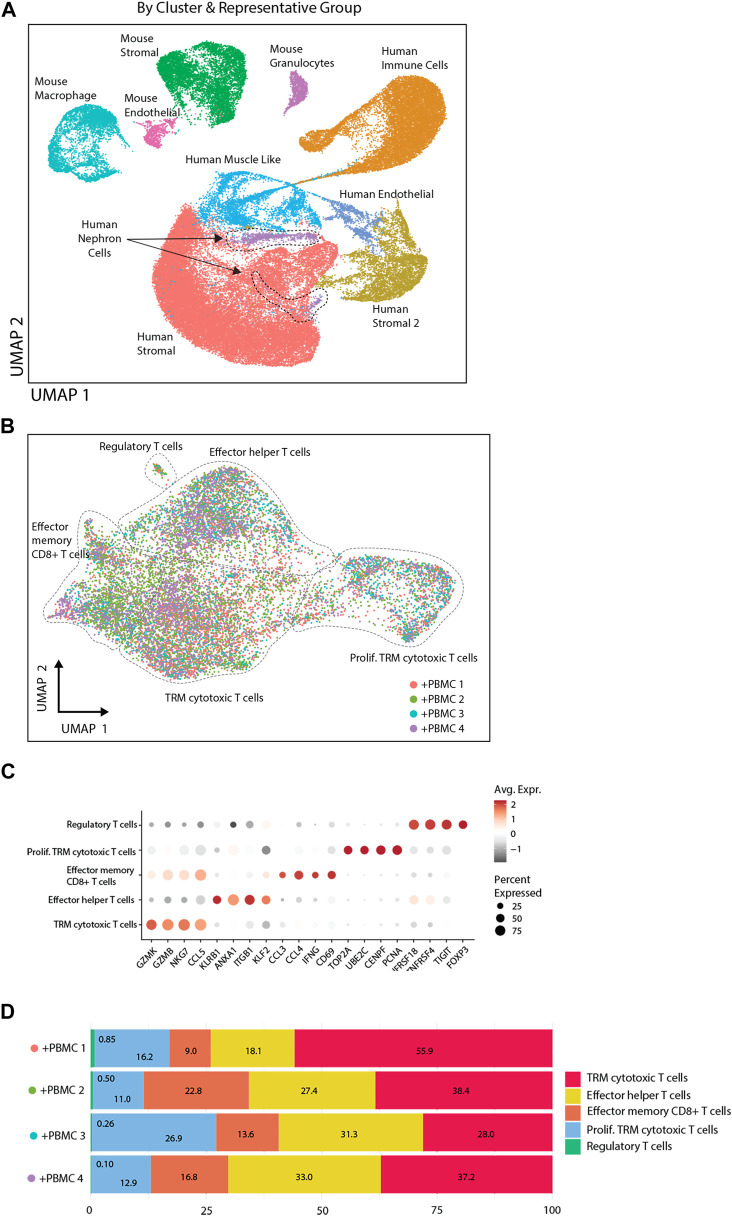
Single cell transcriptomic analysis of implanted kidney organoids. **(A)** UMAP presentation of single cell transcriptomic analysis of organoid cells with and without PBMC by sample and by cell clusters identified on basis of highly variable genes. **(B)** UMAP representation of infiltrated immune cells. Diverse T-cell populations in the organoid are identified by Celltypist. **(C)** Dot plot visualization of T-cell subsets, displaying key markers for the diverse subsets indicated on the X-axis. The size of the dots reflects the frequency of T-cells in each subset that expresses the gene indicated on the X-axis, and the color of the dots reflects the level of expression. **(D)** Frequency distribution of T-cell subsets in four immune cell-infiltrated organoids.

To study the interaction between T-cell subsets and organoids in more detail, single cell transcriptomic analysis of implanted kidney organoids was performed. We analyzed 37,283 cells from 3 control organoids (from 2 iPSC lines) and 31,100 cells from 4 organoids (from the same 2 iPSC lines) infiltrated with PBMC from 3 donors (1 donor was used twice). Unsupervised clustering identified heterogeneous populations of human nephron-, stromal-, endothelial-, and muscle-like cells and various murine cell populations (Figure 5A). Stromal cells were divided in two subpopulations, human stromal and human stromal 2, of which the latter consisted of proliferating cells. These cells were most abundant in immune cell-infiltrated organoids, which exhibited multiple T-cell subpopulations including tissue-resident memory (TRM) cytotoxic T-cells, effector helper T-cells, effector memory CD8^+^ T-cells and regulatory T-cells (Figure 5B), and expressed a distinctive pattern of markers (Figure 5C). TRM cytotoxic T-cells made up the larges proportion of infiltrated immune cells across the 4 samples (28%–56%), whereas the regulatory T-cells made up the smallest fraction (0.10%–0.85%) (Figure 5D).

### Ligand-Receptor Interactions Between Immune Cells and Organoid Cells

**FIGURE 6:**
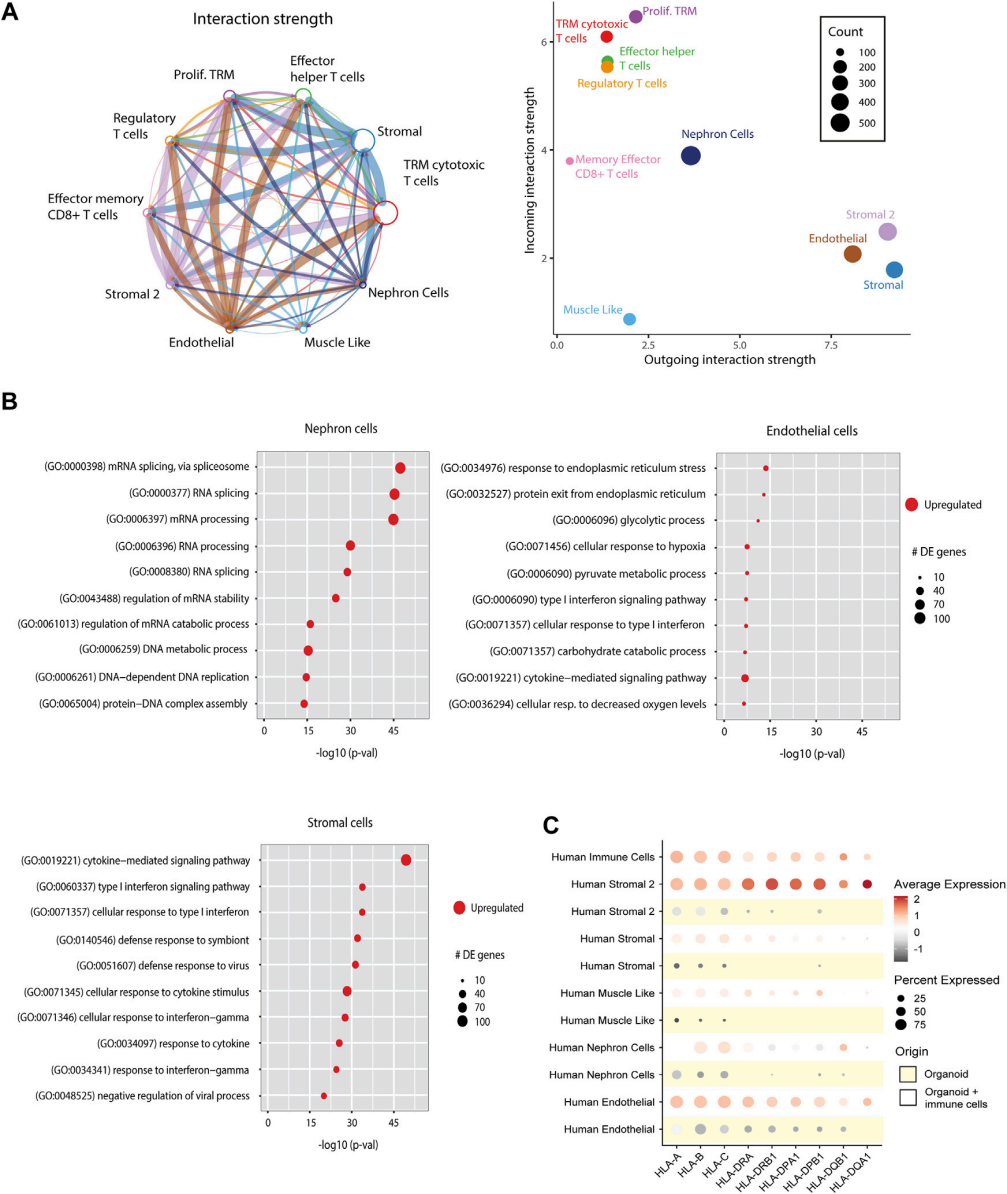
Effect of immune cell infiltration on implanted organoids. **(A)** Ligand-receptor interactions in immune cell-infiltrated organoids. Left: Aggregated cell-cell communication network between all human cells from the immune cell-infiltrated organoids. Circle size denotes number of cells in each group; width of lines denotes total interaction strength between cell groups. Right: Scatter plot showing the dominant senders (ligands) and receivers (receptors). Dot size denotes total number of inferred links. Axis measure the total outgoing or incoming communication strength associated with each cell cluster. **(B)** Differentially expressed (DE) pathways between nephron cells, stromal cells and endothelial cells from control organoids vs. organoids infiltrated with immune cells, demonstrating upregulation of immune response pathways and metabolic pathways upon immune cell infiltration. Dot size indicates number of differentially expressed genes within the pathways. **(C)** Dot plot visualization comparing the expression of HLA genes in all cell types of kidney organoids with and without immune cells. Each dot represents the averaged relative gene expression of 4 organoids.

To examine the cross-talk between immune cells and cells of the organoids, we performed ligand-receptor interaction analysis on immune cell-infiltrated organoids (Figure 6A). The strongest interactions were found between immune cell receptors and endothelial and stromal cell population ligands. The ligand-receptor interactions were involved in cell adhesion pathways (CD44, CD47, CD6, and collagens), antigen presentation (CD74), and inflammatory responses (ITGA, ITGB, GZMA, and TNFSF9) (Supplementary Figure S7).

### Immune Cells Affect Molecular Pathways in Organoids

We next compared pathways in immune cell-infiltrated organoids with control organoids. Nephron cells from immune cell-infiltrated organoids showed an upregulation of metabolic pathways, and endothelial cells showed upregulated stress response pathways. Stromal cells showed an upregulation of diverse immune response pathways (Figure 6B). The immune activated status of stromal cell populations was further characterized by the strongly upregulated expression of HLA genes (Figure 6C). Such upregulation of HLA was also observed in endothelial cells, albeit at a lesser level, and was hardly detectable in nephron cells.

### Immune Cells Affect Maturation and Health Status of Organoids

**FIGURE 7:**
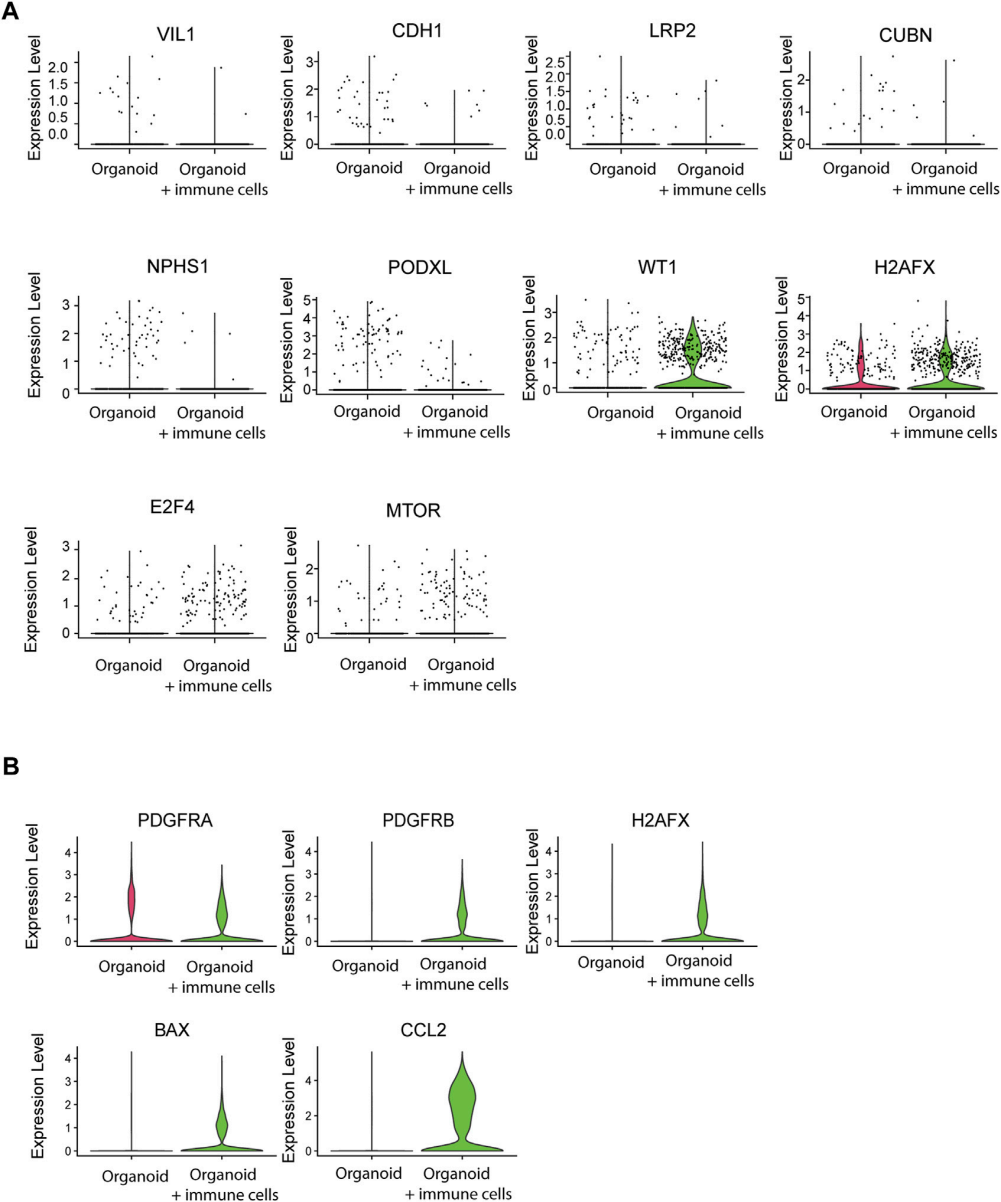
Effect of immune cell infiltration on gene expression of nephron structures and stromal cells. **(A)** Single cell mRNA expression analysis of tubular structural and functional markers VIL1, CDH1, LRP2 and CUBN, the glomerular markers NPHS1, PODXL and WT1, the DNA damage marker H2AFX and the stress response markers E2F4 and MTOR in nephron cells from organoids with and without infiltrated immune cells. **(B)** Single cell mRNA expression analysis of stromal markers PDGFRA and PDGFRB, the DNA damage/injury markers H2AFX and BAX and the immune response marker CCL2 in stromal cells from organoids with and without infiltrated immune cells.

Finally, we examined whether immune cells affected organoid maturation through analysis of expression of structural marker genes. Tubular cells downregulated VIL1, CDH1, LRP2, and CUBN and glomerular cells downregulated NPHS1 and PODXL1 in the presence of infiltrated immune cells, while WT1 was increased (Figure 7A). Furthermore, the DNA damage marker H2AFX and the activation markers E2F4 and MTOR were increased. Stromal cells displayed elevated expression of the stromal markers PDGFRA and PDGFRB, the injury and stress response genes H2AFX and BAX, and of the immune response gene CCL2 (Figure 7B). Together, these results demonstrate that infiltration of adaptive immune cells influences the maturation and health status of kidney organoids.

## Discussion

We demonstrated robust infiltration of immune cells upon *in vitro* and *in vivo* exposure of kidney organoids to immune cells. We found differences in the subsets of immune cells that invaded kidney organoids, and in their localization within the organoids between the *in vitro* and *in vivo* models. Monocytic cells were the most dominant cell type to invade kidney organoids upon co-culture with PBMC, followed by CD4^+^ T-cells and CD8^+^ T-cells, while *in vivo* organoids were infiltrated predominantly by CD8^+^ T-cells, followed by CD4^+^ T-cells*. In vitro*, T-cells showed a preference for nephron structures, while *in vivo* T-cells were mostly found in stromal areas. These differences are likely caused by differences in immune cell composition and organoid structure between the two models. In the animal model, T-cells undergo massive proliferation while other immune cell types show poor survival. After 4 weeks, the CD4/CD8 T-cell ratio is skewed towards CD8^+^ T-cells and this is reflected in the abundant CD8^+^ T-cell infiltration in the organoids. Differences in organoid composition between the *in vitro* and *in vivo* models likely affect the localization of immune cells. Implanted organoids are 8 weeks older and display an expansion of stromal cells. The stroma is furthermore activated, characterized by high expression of HLA, which attracts T-cell infiltrates, and the route of entry for immune cells is different between the models, as implanted organoids are vascularized, while *in vitro* organoids are exposed to immune cells under static submerged conditions. There were also differences in pro-fibrotic and inflammatory transcriptional profiles between *in vitro* and *in vivo* organoids. *In vitro* organoids showed mild inflammatory responses upon immune cell infiltration compared to *in vivo* organoids, which may be a result from the shorter exposure to immune cells (1 week vs. 4 weeks) and from the fact that the conditions in the *in vivo* model drive expansion of the T cells, which results in activation of the cells. In contrast, PBMC used in the *in vitro* experiments were kept in resting conditions where the organoids were the only activation trigger. The *in vitro* organoids showed a clear pro-fibrotic response upon exposure to immune cells. Such a fibrotic response was not seen *in vivo*, but this is explained by the already abundant fibrosis in the control organoids driven in part by the higher age of the organoids. In addition, host derived factors potentially play a role in fibrosis development too. Thus, the *in vitro* and *in vivo* models each have their merit for studying mechanisms of interaction with immune cells.

We demonstrated that immune cells invade interstitial tissue and nephron structures of kidney organoids *in vitro* and after implantation. This is a pathophysiological process that reflects the usability of kidney organoids to model transplant rejection. Immune cell invasion affected kidney organoid differentiation status, but did not result in complete destruction of kidney organoids. Glomerular and tubular structures were still present 4 weeks after administration of immune cells, albeit in an injured state. We observed that implanted organoids displayed signs of injury such as interstitial fibrosis, but this was not different between organoids with and without immune cells. Implanted organoids are exposed to multiple challenges such as a xeno-environment, hypoxia, vascularization and wound healing processes, which induce injury in organoids independent of the presence of immune cells. We therefore specifically interpreted immune-related injury as the upregulation of proinflammatory receptors and ligands and a downregulation of terminal differentiation markers and functional genes, which we observed in both epithelial and stromal compartments of the infiltrated kidney organoids. Similarly, a study comparing healthy kidney and kidney transplant biopsies using single cell transcriptomic analysis revealed a downregulation of terminal differentiation markers and upregulation of pro-inflammatory genes in biopsy epithelia [23].

T-cell mediated rejection remains a relevant issue in chronic allograft rejection [24]. Previously, the humanized skin transplant model has often been utilized as a model of solid organ transplantation [21]. Our kidney organoid allograft model is better suited to study T-cell-mediated immune responses and the impact of T-cell-infiltration on human kidney-like tissue. We found a diversity of T-cell subsets in the organoid grafts and while it is likely that T-cell infiltration occurred upon antigen-specific activation, it is interesting that CD4^+^ T-cells with a resting phenotype were observed in the grafts. Compared to CD4^+^ T-cells, CD8^+^ T-cells were mostly highly activated and proliferative, which may be triggered through direct recognition of HLA class I present on the organoids. We also detected a unique cluster of T-cells with a tissue resident phenotype, which discloses their long-term presence in the organoids and proves that the organoid microenvironment supports long-term survival of T-cells.

A limitation of the study is a lack of analysis of the composition of the PBMC used in the *in vitro* and *in vivo* studies. Characterization of the PBMC composition before exposure to kidney organoids would have allowed a longitudinal analysis of the effects of kidney organoids on different cell populations. Furthermore, in the present study we were not able to HLA type PBMC and kidney organoids, and therefore the degree of HLA mismatching between organoids and PBMC could not be correlated to the severity of the inflammatory response. However, whereas the degree of HLA mismatching is correlated with the risk for rejection of kidney transplants over large numbers, such correlation cannot be expected for small numbers of donor-recipient pairs as the immunogenicity of individual HLA mismatches is highly variable. Another limitation of the study is the immature status of kidney organoids, which could affect the type of infiltrating immune cells and their activity in the tissue. To compare the infiltration of T cells in kidney organoids with that of adult kidney tissue, T cell infiltration could have been analyzed in the native mouse kidneys. However, this would imply studying a xeno-response, which is intrinsically different from human allo-immunity, and we therefore refrained from analyzing native kidneys.

In the present *in vivo* studies, kidney organoids were implanted subcutaneously. Earlier studies demonstrated successful engraftment of kidney organoids implanted under the kidney capsule [4, 7]. While for future replacement therapies kidney organoids will need to be connected to a urine collection system, for current research questions is it important to achieve efficient vascularization and maturation of kidney organoids, which occurs in both models. The advantage of the subcutaneous model is that it allows implantation of multiple organoids per animal and it requires less invasive procedures.

In conclusion, we characterized the heterogeneous interaction of immune cells with iPSC-derived kidney organoids. Introducing immune cells in kidney organoid creates a more realistic model for studying kidney disease and drug interactions, and allows alloreactivity studies. This will ultimately advance the utility of kidney organoids in regenerative medicine.

## Data Availability

The datasets presented in this study can be found in online repositories. The names of the repository/repositories and accession number(s) can be found below: https://www.ncbi.nlm.nih.gov/, GSE211803.

## Supplementary Material

SUPPLEMENTARY FIGURE S1 Representative genes used for identification of non-immune cell clusters in single cell RNA-sequenced kidney organoids.

SUPPLEMENTARY FIGURE S2 Validation of the in vivo model. **(A)** Spleen weights in mice that received PBMC + organoid, PBMC only, and organoid only (*n* = 10 mice PBMC + organoid, *n* = 5 PBMC only, *n* = 8 organoid only from 3 individual experiments). **(B)** Proportion of live CD45+ human and mouse cells in blood (*n* = 16 mice with PBMC + organoid, *n* = 7 PBMC only, *n* = 14 organoid only, from 5 individual experiments) and spleen (*n* = 15 mice with PBMC + organoid, *n* = 6 PBMC only, *n* = 9 organoid only from 4 individual experiments) determined by flow cytometry. **(C)** Proportion of CD4+ and CD8+ T-cells in the blood (*n* = 16 mice with PBMC + organoid and *n* = 7 PBMC only from 5 individual experiments) and spleen (*n* = 16 with PBMC + organoid and *n* = 7 PBMC only from 5 individual experiments) determined by flow cytometry. **(D)** Proportion of live CD45+ human and mouse cells in organoids determined by flow cytometry (*n* = 18 organoids with PBMC from 11 mice and *n* = 5 organoids only from 4 mice from 4 individual experiments).

SUPPLEMENTARY FIGURE S3 Representative flow cytometry plots of live human and mouse CD45^+^ leukocytes in the blood and spleen of mice after adoptive transfer of human PBMC.

SUPPLEMENTARY FIGURE S4 Representative flow cytometry plots of live human CD3^+^CD4^+^ and CD3^+^CD8^+^ T-cells in CD45^+^ leukocytes in the blood and spleen of mice after adoptive transfer of human PBMC.

SUPPLEMENTARY FIGURE S5 Representative flow cytometry plots of live human CD45^+^CD3^+^CD4^+^ and CD45^+^CD3^+^CD8^+^ T-cells in implanted organoids.

SUPPLEMENTARY FIGURE S6 Representative flow cytometry plot of CD4^+^ and CD8^+^ tissue-resident memory T-cells in implanted organoids.

SUPPLEMENTARY FIGURE S7 Ligand-receptor interactions between organoid cells and immune cells. Dot plots showing top significant interactions (ligand-receptor pairs) from organoid cells to immune cells, and immune cells to organoid cells. Dot color and size represent the calculated communication strength for each pair, and its *p*-value.

SUPPLEMENTARY TABLE S1 Details of the used antibodies.

SUPPLEMENTARY TABLE S2 Complete list of genes used for identification of non-immune cell clusters.
