# Supplementary figures and images for: Interactions of the Immune System with Human Kidney Organoids
Source: Transpl Int. 2024 Apr 18;37:12468. doi: 10.3389/ti.2024.12468 (PMC11064018; doi:10.3389/ti.2024.12468)

Supplementary figure 7

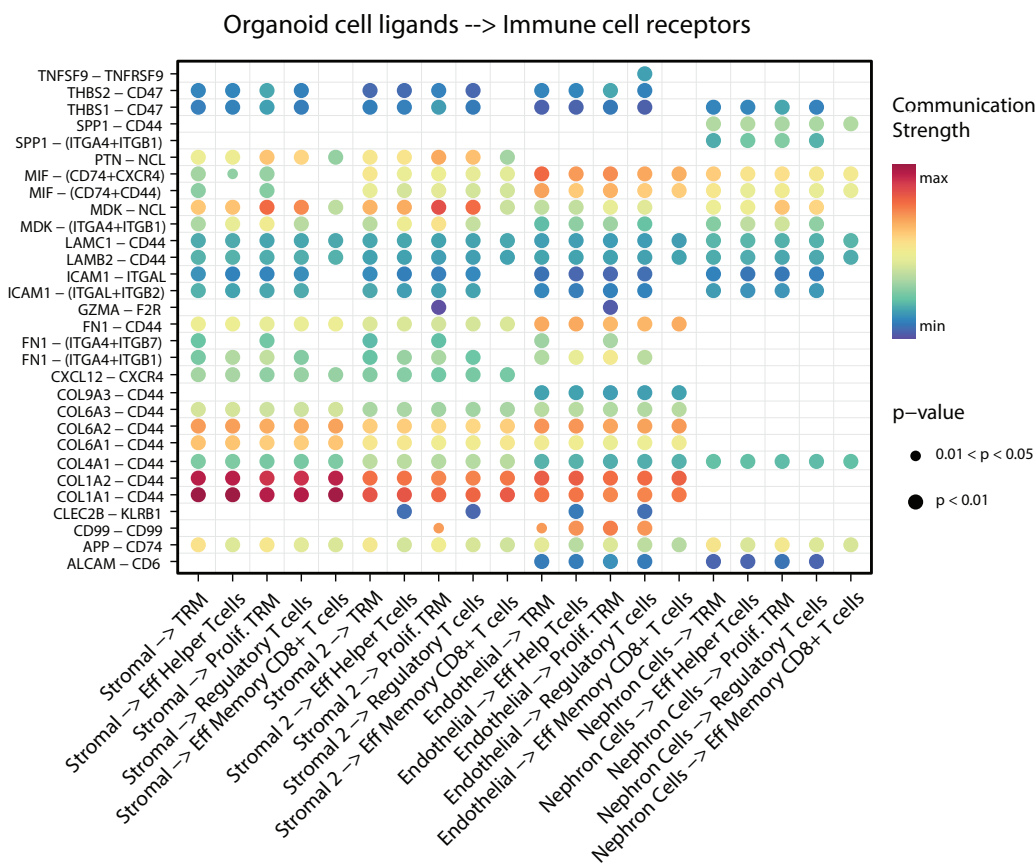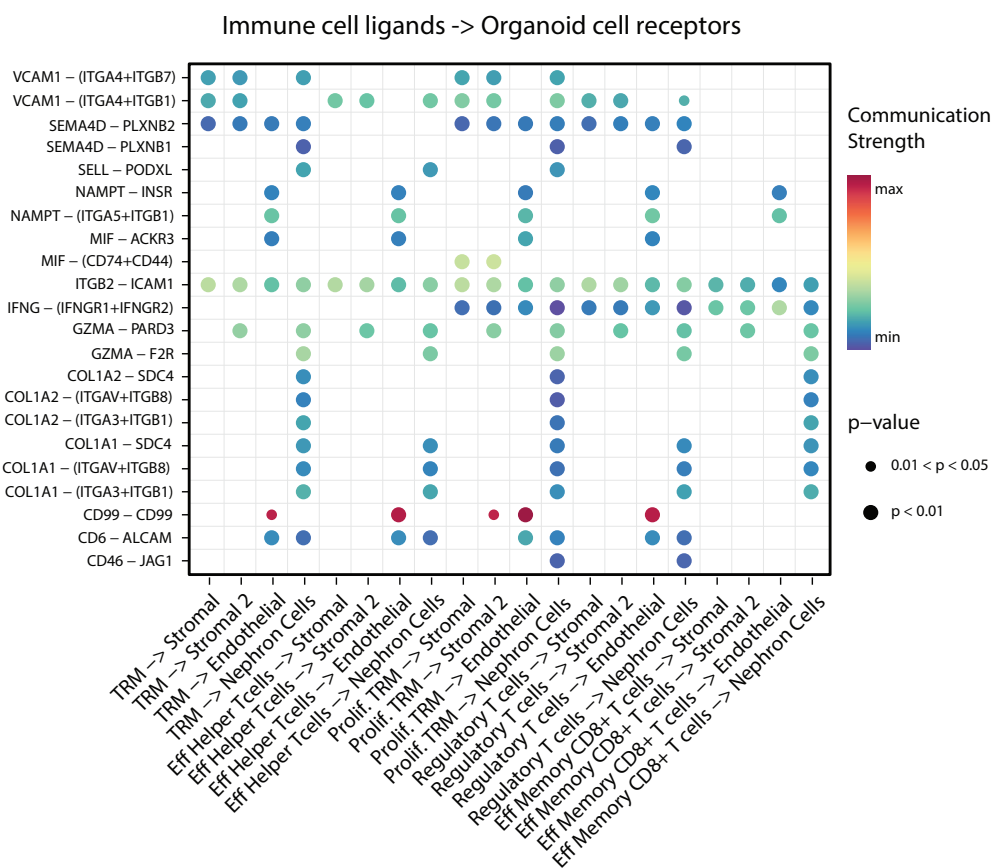

Supplement: Supplementary file 1 [file DataSheet7.PDF]

Supplementary Figure 2

A

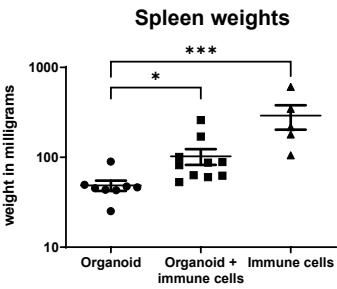

B

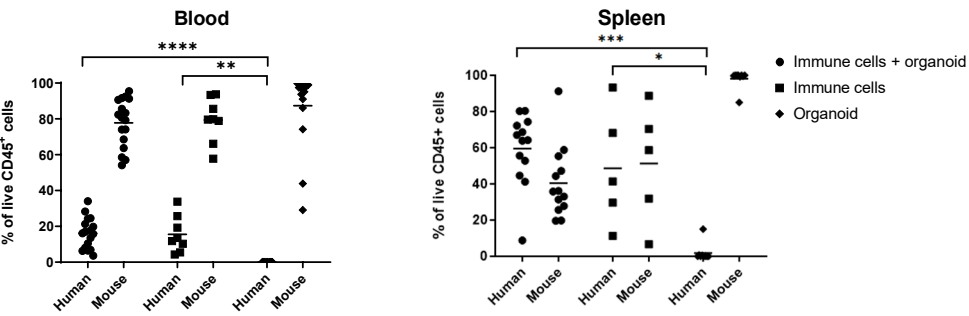

C

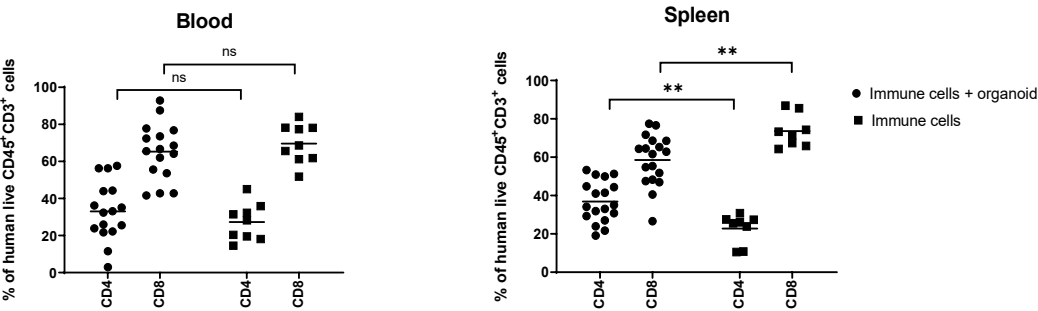

D

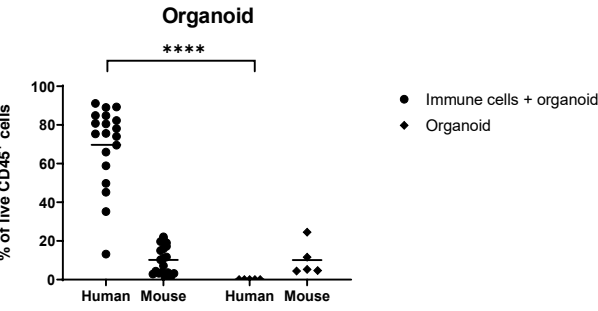

Supplement: Supplementary file 2 [file DataSheet2.PDF]

Supplementary figure 1

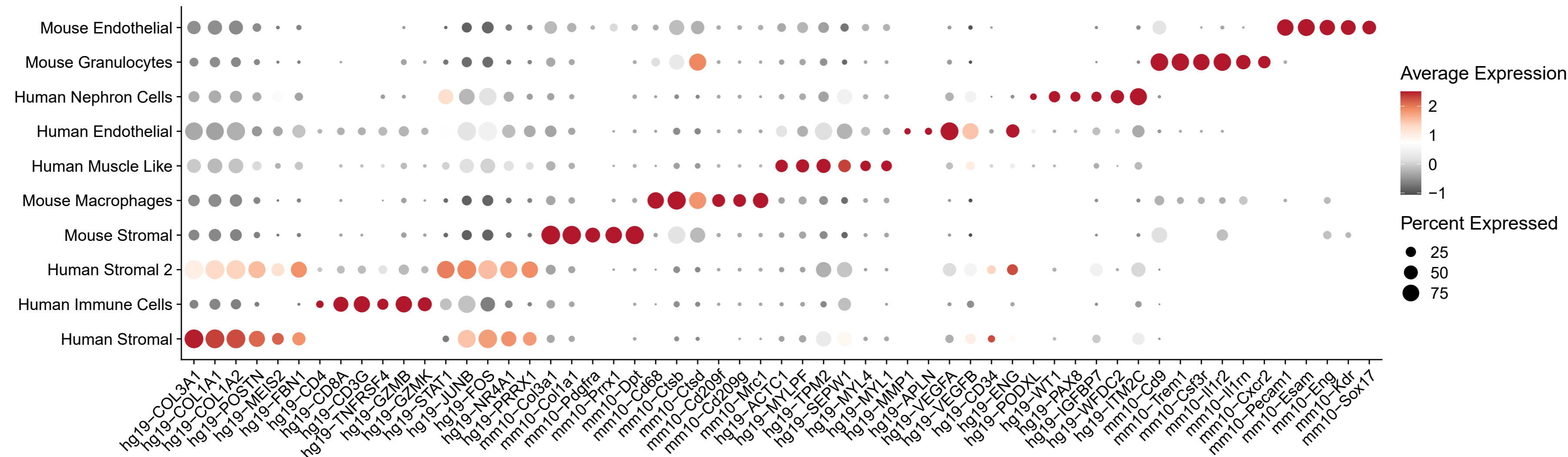

Supplement: Supplementary file 8 [file DataSheet1.PDF]

Supplementary figure 5

Organoid 4 weeks after human PBMC administration

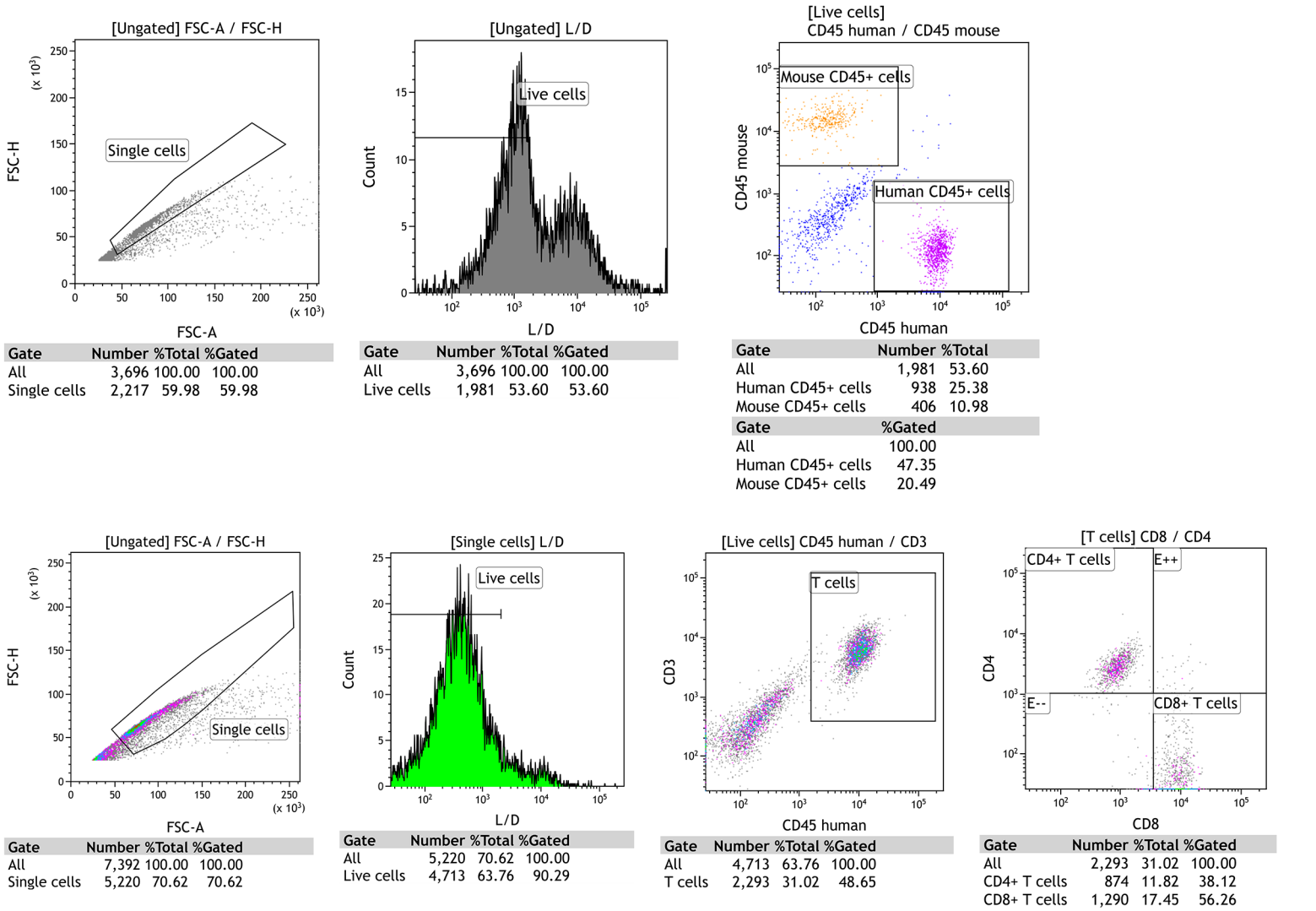

Supplement: Supplementary file 9 [file DataSheet5.PDF]
