# Supplementary material for: Interactions of the Immune System with Human Kidney Organoids
Source: Transpl Int. 2024 Apr 18;37:12468. doi: 10.3389/ti.2024.12468 (PMC11064018; doi:10.3389/ti.2024.12468)
Supplement: Supplementary file 3 [file DataSheet4.PDF]

Supplementary figure 4

Mouse blood 4 weeks after human PBMC administration

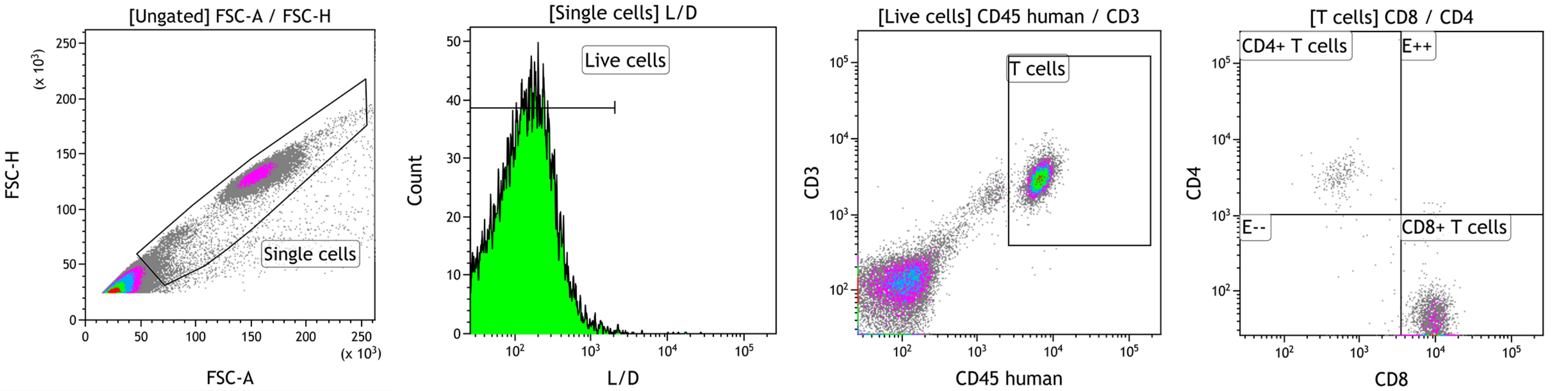

Gate	Number	%Total	%Gated
All	88,484	100.00	100.00
Single cells	22,280	25.18	25.18

Gate	Number	%Total	%Gated
All	22,280	25.18	100.00
Live cells	22,251	25.15	99.87

Gate	Number	%Total	%Gated
All	22,251	25.15	100.00
T cells	6,921	7.82	31.10

Gate	Number	%Total	%Gated
All	6,921	7.82	100.00
CD4+ T cells	213	0.24	3.08
CD8+ T cells	6,641	7.51	95.95

Mouse spleen 4 weeks after human PBMC administration

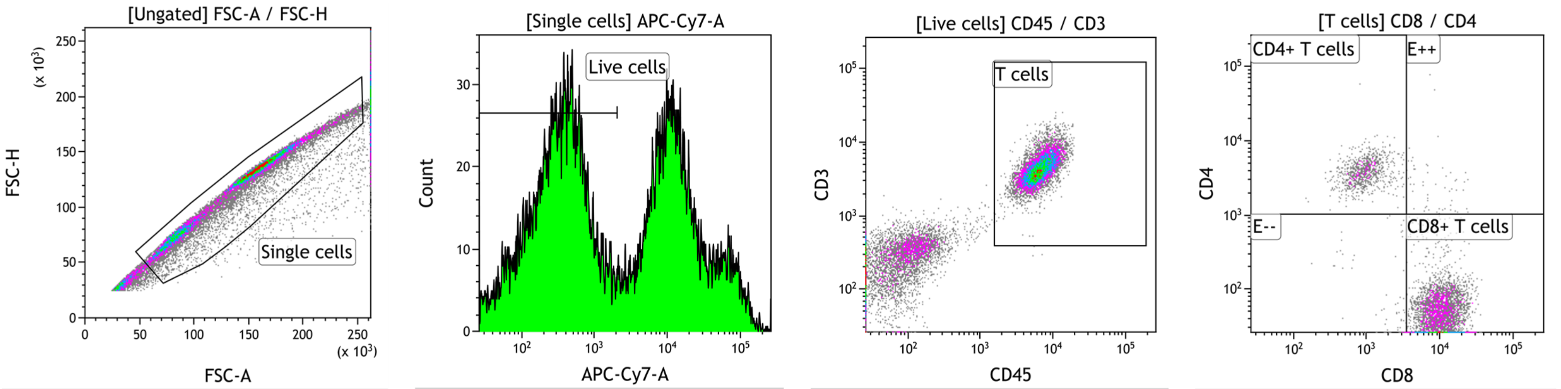

Gate	Number	%Total	%Gated
All	24,342	100.00	100.00
Single cells	19,985	82.10	82.10

Gate	Number	%Total	%Gated
All	19,985	82.10	100.00
Live cells	13,861	56.94	69.36

Gate	Number	%Total	%Gated
All	13,861	56.94	100.00
T cells	8,625	35.43	62.22

Gate	Number	%Total	%Gated
All	8,625	35.43	100.00
CD4+ T cells	904	3.71	10.48
CD8+ T cells	7,590	31.18	88.00
