# Supplementary material for: Interactions of the Immune System with Human Kidney Organoids
Source: Transpl Int. 2024 Apr 18;37:12468. doi: 10.3389/ti.2024.12468 (PMC11064018; doi:10.3389/ti.2024.12468)
Supplement: Supplementary file 4 [file DataSheet6.PDF]

Supplementary figure 6

Mouse Spleen	Kidney organoid

CD69

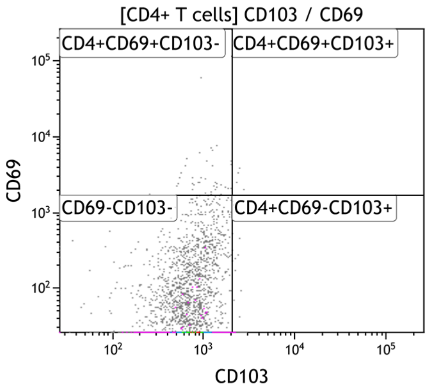

Gate	Number	%Total	%Gated
All	6.134	26,67	100,00
CD4+CD69-CD103+	47	0,20	0,77
CD4+CD69+CD103-	39	0,17	0,64
CD4+CD69+CD103+	4	0,02	0,07
CD69-CD103-	6.044	26,28	98,53

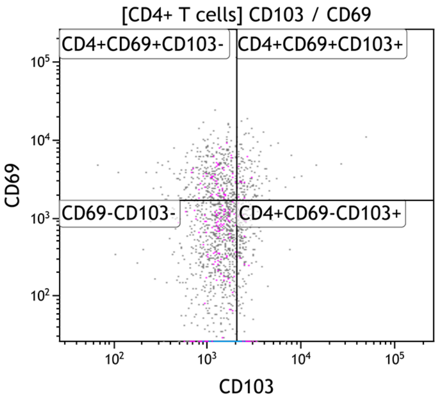

Gate	Number	%Total	%Gated
All	1.767	17,67	100,00
CD4+CD69-CD103+	256	2,56	14,49
CD4+CD69+CD103-	396	3,96	22,41
CD4+CD69+CD103+	141	1,41	7,98
CD69-CD103-	974	9,74	55,12

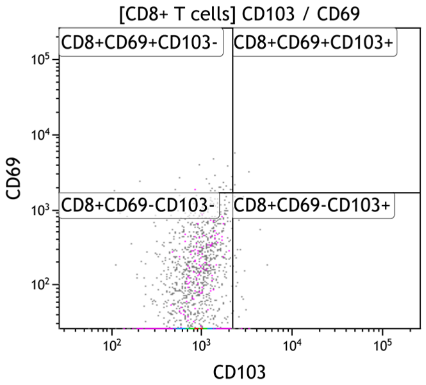

Gate	Number	%Total	%Gated
All	3.458	15,03	100,00
CD8+CD69-CD103-	3.399	14,78	98,29
CD8+CD69-CD103+	32	0,14	0,93
CD8+CD69+CD103-	23	0,10	0,67
CD8+CD69+CD103+	4	0,02	0,12

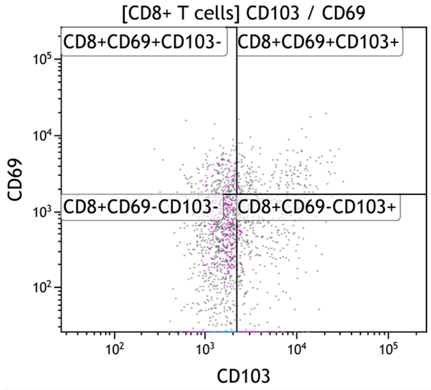

Gate	Number	%Total	%Gated
All	2.004	20,04	100,00
CD8+CD69-CD103-	1.125	11,25	56,14
CD8+CD69-CD103+	473	4,73	23,60
CD8+CD69+CD103-	215	2,15	10,73
CD8+CD69+CD103+	191	1,91	9,53

CD103
