# Supplementary material for: Interactions of the Immune System with Human Kidney Organoids
Source: Transpl Int. 2024 Apr 18;37:12468. doi: 10.3389/ti.2024.12468 (PMC11064018; doi:10.3389/ti.2024.12468)
Supplement: Supplementary file 5 [file Table1.pdf]

Supplementary table 1

Antibody	Type	Concentration	Company	Clone	Procedure	Ab incubation time
Villin	Rabbit	1/800	Abcam	EPR3491	Optiview CC1 32'	32 minutes
ECAD	Mouse	0.314 µg/ml	Ventana	36	Optiview CC1 32'	16 minutes
WT1	Mouse	2.49 µg/ml	Cell Marque	6F-H2	Optiview CC1 56'	32 minutes
Granzyme B	Mouse	0.4 µg/ml	Cell Marque	EPR230	Optiview CC1 32'	32 minutes
Ki67	Rabbit	0.4 µg/ml	Ventana	30-9	Ultraview CC1 36'	28 minutes
CD3	Rabbit	0.4 µg/ml	Ventana	2GV6	Optiview CC1 32'	32 minutes
CD4	Rabbit	2.5 µg/ml	Ventana	SP35	Ultraview CC1 64'	8 minutes
CD8	Rabbit	0.35 µg/ml	Ventana	SP57	Optiview CC1 16'	32 minutes
CD20	Mouse	0.3 µg/ml	Ventana	L2G	Ultraview CC1 64'	44 minutes
CD45	Mouse	8.31 µg/ml	Ventana	2B11	Optiview CC1 32'	4 minutes
CD68	Mouse	0.4 µg/ml	Ventana	KP1	Optiview CC1 16'	8 minutes
CD163	Mouse	0.23 µg/ml	Ventana	MRQ-26	Optiview CC1 32'	20 minutes
FOXP3	Mouse	0.5 mg/ml	Thermofisher	236A/E7	Optiview CC1 32'	32 minutes
HLA-DR	Mouse	1/400	DAKO	CR3/43	Ultraview CC1 64'	32 minutes
PD1	Mouse	2.84 µg/ml	Ventana	NAT105	Optiview CC1 16'	20 minutes
PDL1	Rabbit	1.61 µg/ml	Ventana	SP263	Optiview CC1 64'	16 minutes
Kim1	Mouse	1/400	R&D systems	219211	Optiview CC1 32’	32 minutes
Col1A1	Rabbit	1/3000	Abcam	EPR7785	Optiview CC1 32’	32 minutes
