# Supplementary material for: Interactions of the Immune System with Human Kidney Organoids
Source: Transpl Int. 2024 Apr 18;37:12468. doi: 10.3389/ti.2024.12468 (PMC11064018; doi:10.3389/ti.2024.12468)
Supplement: Supplementary file 6 [file Table2.pdf]

Supplementary table 2

Cell Type	Human cells				
	Loop of Henle	Tubular cells	Podocytes	Stromal 1	Stromal 2
Genes	hg19-SLC12A1	hg19-TSPAN12	hg19-TCF21	hg19-MEIS2	hg19-MEIS2
	hg19-WFDC2	hg19-IGFBP7	hg19-PODXL	hg19-COL3A1	hg19-COL3A1
	hg19-KCNJ1	hg19-MPC2	hg19-VEGFA	hg19-COL1A2	hg19-COL1A2
	hg19-TFCP2L1	hg19-HNF1B	hg19-WT1	hg19-EDNRA	hg19-EDNRA
	hg19-CPS1	hg19-SLC27A2	hg19-SYNPO	hg19-PRRX2	hg19-PRRX2
	hg19-ITM2C	hg19-PAX8	hg19-PTPRO	hg19-IGF1	hg19-IGF1
	hg19-TMPRSS4	hg19-CUBN	hg19-NPHS2	hg19-PRRX1	hg19-PRRX1
	hg19-AFP	hg19-PSMA2	hg19-ITIH5	hg19-THY1	hg19-THY1
	hg19-MECOM	hg19-BNIP3	hg19-ANXA1	hg19-MEOX1	hg19-MEOX1
	hg19-CLDN10	hg19-GLYATL1	hg19-GPX3	hg19-NT5E	hg19-NT5E
	hg19-DUSP9	hg19-SPP1	hg19-CLIC5	hg19-PDGFRA	hg19-PDGFRA
	hg19-MAL	hg19-AFP	hg19-NPHS1	hg19-PDGFRB	hg19-PDGFRB
	hg19-TFAP2B	hg19-PDZK1	hg19-MYL9	hg19-COL1A1	hg19-COL1A1
	hg19-CLU	hg19-SMIM24	hg19-MAFB	hg19-FGFBP2	hg19-FGFBP2
	hg19-ACPP	hg19-SLC22A8		hg19-FGFBP1	hg19-FGFBP1
	hg19-LDHB	hg19-CALB1		hg19-MEIS2	hg19-MEIS2
	hg19-CA12	hg19-ALDH1A1			
	hg19-ACSL4	hg19-MAL			
	hg19-TUBB2B	hg19-LRP2			
	hg19-CLCN5	hg19-SLC3A1			
		hg19-VIL1			
		hg19-CDH1			
		hg19-MUC1			
		hg19-UMOD			
		hg19-CDH16			
		hg19-ESRRG			
		hg19-ERBB4			
		hg19-DEFB1			
		hg19-TMEM52B			
		hg19-SLC12A1			
		hg19-GATA3			
		hg19-MECOM			
		hg19-WFDC2			
		hg19-POU3F3			
		hg19-SLC9A3			
		hg19-LRP2			
		hg19-MUC1			

Human cells		Mouse Cells			
**Muscle like**	**Endothelial**	**Macrophages**	**Endothelial**	**Stromal**	**Granulocytes**
hg19-ACTC1	hg19-PECAM1	mm10-Itgam	mm10-Pecam1	mm10-Meis2	mm10-Cd9
hg19-MYLPF	hg19-CD34	mm10-Cd14	mm10-Cd34	mm10-Col3a1	mm10-Trem1
hg19-MYOG	hg19-MMP1	mm10-Cd40	mm10-Mmp1	mm10-Col1a2	mm10-Csf2rb
hg19-MYL1	hg19-ESAM	mm10-Cd64	mm10-Esam	mm10-Ednra	mm10-Csf3r
hg19-MYL4	hg19-ESM1	mm10-Cd68	mm10-Esm1	mm10-Prrx2	mm10-Il1r2
hg19-TNNT1	hg19-APLN	mm10-Tlr7	mm10-Apln	mm10-Igf1	mm10-Il1rn
hg19-PDLIM3	hg19-ENG	mm10-Tom1	mm10-Eng	mm10-Prrx1	mm10-Cxcr2
hg19-MRLN	hg19-KDR	mm10-Fdg4	mm10-Kdr	mm10-Thy1	mm10-Il13ra1
hg19-MYOD1	hg19-ANGPT2	mm10-Ctsb	mm10-Angpt2	mm10-Meox1	mm10-Cd9
hg19-TNNI1	hg19-SOX17	mm10-Ctsd	mm10-Sox17	mm10-Nt5e	
hg19-CKB	hg19-CDH5	mm10-Ctsl	mm10-Cdh5	mm10-Pdgfra	
hg19-USP18		mm10-Cd4		mm10-Pdgfrb	
hg19-NNAT		mm10-Cd8a		mm10-Col1a1	
hg19-TPM2		mm10-Cd3g		mm10-Fgfbp2	
hg19-PFN1		mm10-Cd209f		mm10-Fgfbp1	
hg19-RPS2		mm10-Cd209g			
hg19-RPL18A					
hg19-NACA					
hg19-RPS5					
hg19-RPS3A					
hg19-TAGLN					
hg19-ACTA2					
hg19-SEPW1					
