# Supplementary material for: Interactions of the Immune System with Human Kidney Organoids
Source: Transpl Int. 2024 Apr 18;37:12468. doi: 10.3389/ti.2024.12468 (PMC11064018; doi:10.3389/ti.2024.12468)
Supplement: Supplementary file 7 [file DataSheet3.PDF]

Supplementary figure 3

Mouse blood 4 weeks after human PBMC administration

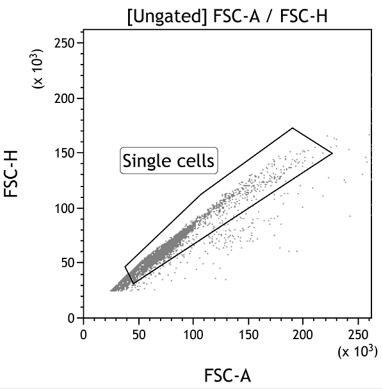

Gate	Number	%Total	%Gated
All	11,012	100.00	100.00
Single cells	9,989	90.71	90.71

Mouse Spleen
Only adoptive transfer

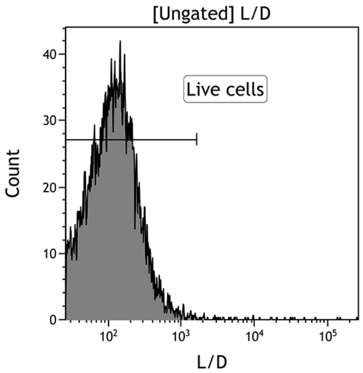

Gate	Number	%Total	%Gated
All	11,012	100.00	100.00
Live cells	10,973	99.65	99.65

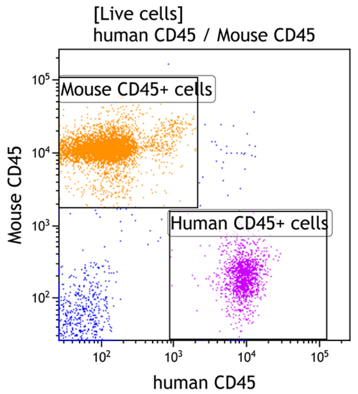

Gate	Number	%Total
All	10,973	99.65
Human CD45+ cells	1,505	13.67
Mouse CD45+ cells	7,968	72.36

Gate	%Gated
All	100.00
Human CD45+ cells	13.72
Mouse CD45+ cells	72.61

Mouse spleen 4 weeks after human PBMC administration

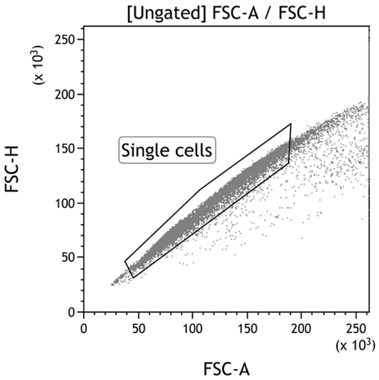

Gate	Number	%Total	%Gated
All	17,151	100.00	100.00
Single cells	14,137	82.43	82.43

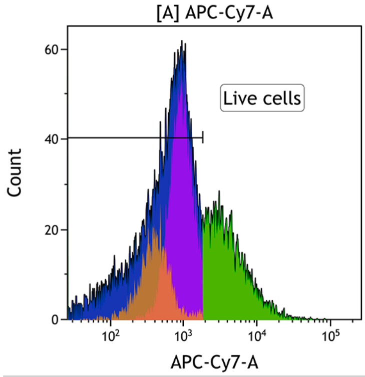

Gate	%Total
All	82.39
Live cells	61.44

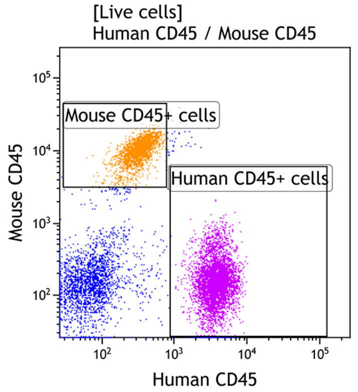

Gate	Number	%Total
All	10,537	61.44
Human CD45+ cells	4,989	29.09
Mouse CD45+ cells	2,337	13.63

Gate	%Gated
All	100.00
Human CD45+ cells	47.35
Mouse CD45+ cells	22.18
